# The Role of Neonatal Ward Environment as a Reservoir for the Dissemination of Multidrug‐Resistant *Klebsiella pneumoniae* in Algeria

**DOI:** 10.1002/mbo3.70346

**Published:** 2026-06-21

**Authors:** Meriem Derkaoui, Martin Alexander Fischer, Mohammed Sebaihia, Rachida Namoune, Fatiha Bouheudjeur, Meryem Beloukarif, Bakhta Madaoui, Bruno Silvester Lopes

**Affiliations:** ^1^ Department of Biology, Laboratory of Molecular Biology, Genomics and Bioinformatics, Faculty of Nature and Life Sciences University Hassiba Benbouali Chlef Algeria; ^2^ Department of Infectious Diseases, Division of Nosocomial Pathogens and Antimicrobial Resistances Robert Koch Institute Wernigerode Germany; ^3^ Public Hospital Establishment Chlef Algeria; ^4^ School of Health and Life Sciences, Life Sciences Teesside University Middlesbrough UK; ^5^ National Horizons Centre Teesside University Darlington UK

**Keywords:** Algeria, antibiotic resistance, hospital environment, K. pneumoniae, ST13, ST8932

## Abstract

*Klebsiella pneumoniae* is a major cause of hospital‐acquired infections and contributes to high mortality. We examined the molecular epidemiology, antibiotic resistance, and virulence features of *K. pneumoniae* from the neonatal ward environment of a hospital in Chlef, Algeria, using whole‐genome sequencing (WGS). Antibiotic susceptibility was assessed with the Vitek 2 automated system (AST‐N365 card). Genomic DNA was extracted with the GF‐1 kit, and WGS was performed by GENEWIZ Europe using the NEBNext Ultra II DNA Library Prep Kit. Species identification and virulence genes were determined using Kleborate, antimicrobial resistance genes were detected with AMRfinder Plus. From 9790 publicly available *K. pneumoniae* genomes, the 112 closest matches were selected, and core‐genome MLST and a minimum‐spanning tree were generated in SeqSphere + v10.5.04 using a MLST and cgMLST scheme. All isolates were multidrug‐resistant, and WGS identified 22 resistance genes. Core genes detected across all samples included the *bla*_CTX‐M‐15_, *bla*_TEM‐1_, *oqxA, oqxB, aph(6)‐Id, aph(3”)‐Ib, sul2, fosA5, emrD* and *kdeA*. Other genes, including *bla*_SHV‐1_, *bla*_SHV‐11_, *bla*_DHA‐1_, *bla*_OXA‐1_, *qnrB4, qnrS1* and *aac(6′)‐Ib‐cr5* were also observed. Yersiniabactin‐associated virulence genes *ybt* and *irp* were detected in KP32, KP37 and KP57 (high risk clone ST13) while *qacE* was present only in KP55 (novel strain ST8932). This study highlights the persistence of *bla*_CTX‐M‐15_, *qnr*, and *bla*_DHA‐1_ in *K. pneumoniae* from the neonatal ward environment. The presence of the high‐risk strain ST13 is particularly concerning, as its identical allelic profiles indicate likely clonal spread within the hospital. These results suggest the hospital environment may act as a reservoir for multidrug‐resistant *K. pneumoniae*.

## Graphical abstract

Whole‐genome sequencing of multidrug‐resistant *Klebsiella pneumoniae* from a neonatal ward environment in Chlef revealed persistent resistance genes and the clonal spread of high‐risk ST13. These findings highlight the hospital environment as a potential reservoir for transmission of resistant *K. pneumoniae*.

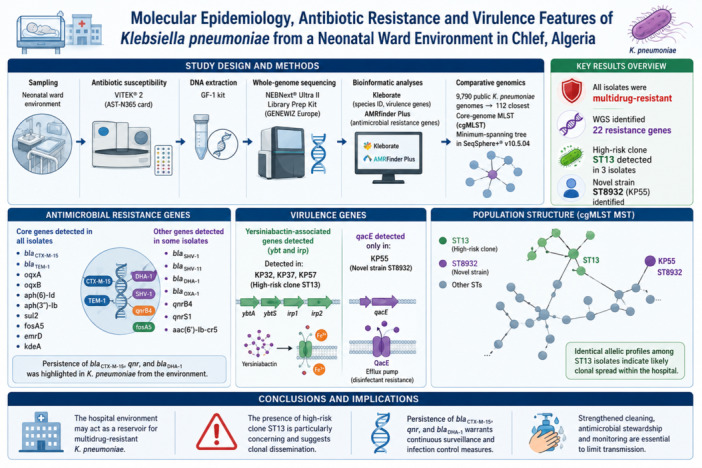

## 1 Introduction

*K. pneumoniae* is an opportunistic pathogen widely distributed in the environment, including water and soil (Jelić et al. 2019; Zhang et al. 2023). It is a common colonizer of the human gastrointestinal tract that serves as a reservoir for this bacterium, leading to extra‐intestinal infections (Martin et al. 2016; Gorrie et al. 2017). *K. pneumoniae* is also known to be resistant to several families of antibiotics, particularly β‐lactams, (Arato et al. 2021) and has been included on the World Health Organization list of critical priority bacterial pathogens to guide the research and development of new antimicrobial agents (Sati et al. 2025).

A recent study estimated the global burden of *K. pneumoniae* infections and associated antibiotic resistance using data from the Global Burden of Disease Study 2019 and the Global Burden of Antimicrobial Resistance 2019 study (Murray et al. 2022; Ikuta et al. 2022; Song et al. 2025). They modeled 471 million records and estimated approximately 800,000 deaths due to *K. pneumoniae* infections worldwide, of which nearly 80% were attributed to antibiotic resistance. It was observed that the most significant mortality burden occurred in lower‐respiratory‐tract, bloodstream, and intra‐abdominal infections, with approximately 90% of *K. pneumoniae*‐related deaths. Regionally, South Asia and sub‐Saharan Africa were show to be the most impacted, with sub‐Saharan Africa having the highest rate of antibiotic resistance‐linked deaths (> 50%) linked to carbapenem and third‐generation cephalosporin resistance (Song et al. 2025).

In addition to being transmitted via the gastrointestinal tract, *K. pneumoniae* can spread in hospitals through contact with contaminated surfaces and objects (Hassan et al. 2019; Valzano et al. 2024). Several studies in Algeria and worldwide have reported the presence of this bacterium in hospital environments, including inanimate surfaces, air, medical devices, and insects such as the German cockroach, contributing to an increase in healthcare‐associated infections (Hassan et al. 2019; Touati et al. 2007; Menasria et al. 2014; Zenati et al. 2017; Silago 2020; Saadi et al. 2022; Valentin et al. 2021; Manageiro et al. 2024; Kindu et al. 2023).

Hospital neonatal wards require special attention due to the highly susceptible nature of newborns whose immune systems are not fully developed, putting them at severe risk of infectious diseases. A recent study has shown that the burden of disease varies by a country's level of socio‐economic development, with 3,634,421 cases of neonatal infectious diseases recorded worldwide in 2021 (Ni et al. 2025).

Significant risk factors for neonatal infections caused by Gram‐negative bacilli include the use of catheters, prolonged hospitalization, low birth weight, prematurity, and invasive procedures such as nasogastric tubes, parenteral nutrition, and mechanical ventilation (Oliva et al. 2021). *K. pneumoniae* is one of these pathogens, and multiple studies have demonstrated the involvement of this bacterium in neonatal outbreaks and sepsis (Sands et al. 2021; De Baat et al. 2023; Osei Sekyere et al. 2024). Neonatal deaths associated with sepsis are a major concern, with antibiotic resistance being one of the leading causes. It is estimated that up to 214,000 neonatal sepsis deaths worldwide could be attributed to resistant pathogens each year (Laxminarayan et al. 2016).

There are previous reports on the isolation of *K. pneumoniae* from hospital environments in Algeria, (Touati et al. 2007; Zenati et al. 2017; Bouguenoun et al. 2016; Boutarfi et al. 2019) but no reports characterizing isolates from the hospital environment, particularly from neonatal wards, using whole genome sequencing (WGS) approaches. Therefore, this study focused on whole‐genome sequencing (WGS) of *Klebsiella pneumoniae* isolates obtained from the neonatal ward environment of a hospital in Chlef province, Algeria. The aim was to assess their virulence characteristics, antibiotic resistance profiles, and molecular epidemiology, in order to improve understanding of the role of environmental isolates in neonatal wards, particularly their resistance patterns and clonal features that may indicate their potential for dissemination.

## 2 Methods

### 2.1 Bacterial Isolates

The present study included four *K. pneumoniae* isolates, isolated in March 2024, from a bathroom sink and floor of a patient's room (*n* = 2), a nurse's hand (*n* = 1) and a mobile phone of another nurse (*n* = 1) in the neonatal ward of a hospital in the Chlef province of Algeria.

### 2.2 Antibiotic Susceptibility Testing

Antibiotic susceptibility testing was performed using the Vitek 2 automated system (Biomérieux) with a Gram‐negative antimicrobial susceptibility testing card, AST‐N365, following the company's standard procedures. Twelve antibiotics belonging to seven classes were tested: β‐lactams (amoxicillin‐clavulanic acid, piperacillin‐tazobactam, cefazolin, cefoxitin, cefotaxime, and ceftazidime), aminoglycosides (gentamicin), fluoroquinolones (ciprofloxacin), nitrofuran (nitrofurantoin), amphenicol (chloramphenicol), diaminopyrimidines‐sulfonamide association (trimethoprim‐sulfamethoxazole), and phosphonic acid (fosfomycin). The isolates were classified as susceptible, intermediate and resistant according to the Clinical and Laboratory Standards Institute, edition 2021, applied by the Pasteur Institute of Algiers. Bacterial isolates that were resistant to three or more antibiotic families were classified as multidrug‐resistant (Magiorakos et al. 2012).

### 2.3 Whole Genome Sequencing

Bacterial genomic DNA was extracted from pure culture using the GF‐1 bacterial DNA extraction kit (Vivantis) according to the manufacturer's instructions. WGS was performed by GENEWIZ Europe, Azenta Life Sciences (Leipzig, Germany).

DNA library preparation and sequencing were performed using the NEBNext Ultra II DNA Library Prep Kit for Illumina (New England Biolabs, Ipswich, MA, USA) following the manufacturer's recommendations. Genomic DNA was fragmented by acoustic shearing using a Covaris LE220 instrument. Fragmented DNA was cleaned up and end‐repaired. Adapters were ligated after adenylation of the 3' ends, followed by enrichment by limited‐cycle PCR. DNA libraries were validated using the 5300 Fragment Analyzer (Agilent Technologies, Palo Alto, CA, USA), and were quantified using Qubit 4.0 Fluorometer. The sequencing libraries were multiplexed and loaded onto the Illumina NovaSeq. 6000 flow cell according to the manufacturer's instructions. The samples were sequenced using a 2×150 Pair‐End (PE) configuration v1.5. Image analysis and base calling were conducted by the NovaSeq Control Software v1.8.1 on the NovaSeq instrument. Raw sequence data (.bcl files) generated from Illumina NovaSeq was converted into fastQ files and de‐multiplexed using Illumina bcl2fastq program version2.20. One mismatch was allowed for index sequence identification.

### 2.4 Genome Assembly, Annotation, and Alignment

Sequence reads were trimmed to remove adapter sequences and nucleotides with poor quality using (Trimmomatic v.0.36) analysis software. Trimmed and quality‐filtered sequences were subsampled 2 × 10^6^ reads per sample and used for genomic assembly using Unicycler v0.5.0 (Wick et al. 2017).

### 2.5 Bioinformatics Analysis

Assemblies were checked for standard QC‐parameters using QUAST contamination using BUSCO (Supporting file 2) (Manni et al. 2021; Gurevich et al. 2013). Mobtyper was used with standard parameters for the binning of contigs of predicted chromosomal and plasmid origin (Robertson et al. 2018). This included a prediction for plasmid MOB‐type Inc‐Type, mobility. Bins were screened for AMR genes using AMRfinder plus to predict chromosomal or plasmid localization of AMR genes (Feldgarden et al. 2021). Species and virulence gene identification were performed using Kleborate (Lam et al. 2021). Core genome multilocus sequence typing (cgMLST) was performed using SeqSphere+ v10.5.04 (Ridome, Münster, Germany) with the 7 loci MLST and 2358 loci cgMLST scheme as described elsewhere (Wahl et al. 2024).

### 2.6 Comparison Dataset Construction

9790 *K. pneumoniae* genomes on the assembly level “complete,” “chromosome” and “scaffold” were downloaded from the NCBI RefSeq. Mash (Ondov et al. 2016) was used to select the *n* = 50 closest matches based on mash distance were selected for each of the two representative Isolates KP55 (as ST8932 isolate) and KP32 (as representative for the closely related ST13 isolates). Isolate assemblies were uploaded to Pathogenwatch (https://pathogen.watch/version 23.5.0, accessed October 1, 2025), and the four phylogenetically closest isolates based on the SNP distance tree (SAMN16721990, SAMEA7190524, SAMN18207166, and SAMN27533720) were added to the comparison dataset (Argimón et al. 2021). In addition, JSpecies (Richter et al. 2016) was used to validate species identification and the eight closest isolates based on average nucleotide identity were added to the comparison dataset (SAMN03076169, SAMN04158310, SAMN04011443, SAMN02581243, SAMEA2738239, SAMN03280387, SAMN03280427, SAMN04011425). After removing duplicate sequences, our final dataset comprised 112 unique isolates from the aforementioned data sources (Supporting file 1). These reference sequences, together with our four study isolates, were used to construct the cgMLST‐based minimal spanning tree in SeqSphere+ (Ridome, Münster, Germany).

## 3 Results

### 3.1 Antibiotic Susceptibility of the Isolates

The results of the minimum inhibitory concentration (MIC) showed a high level of resistance to most antibiotics, with MIC values listed in Table 1. All isolates were resistant to amoxicillin‐clavulanic acid, cefazolin, cefotaxime, ceftazidime, gentamicin, nitrofurantoin, and fosfomycin. Resistance and intermediate resistance were also observed against piperacillin‐tazobactam and ciprofloxacin. However, all our isolates were susceptible to cefoxitin and chloramphenicol, except KP55, which was resistant to cefoxitin (MIC ≥ 64 µg/mL) and intermediate to chloramphenicol (MIC = 16 µg/mL). Trimethoprim‐sulfamethoxazole susceptibility was seen in KP32 and KP57, while KP37 and KP55 were resistant (Table 1). All isolates had the multidrug‐resistant phenotype.

**Table 1:** Source of K. pneumoniae isolates and their minimum inhibitory concentration values.

Isolate	Source	AMC	TZP	CZ	FOX	CTX	CAZ	GM	CIP	F	C	SXT	FOS
KP32	Hand of nurse A	≥ 32 R	64 I	≥ 64 R	≤ 4 S	≥ 64 R	≥ 64 R	≥ 16 R	≥ 4 R	256 R	8 S	40 S	≥ 256 R
KP37	mobile phone of nurse B	≥ 32 R	64 I	≥ 64 R	≤ 4 S	≥ 64 R	≥ 64 R	≥ 16 R	≥ 4 R	256 R	4 S	80 R	≥ 256 R
KP55	Patient room 02 (Bathroom sink)	≥ 32 R	64 I	≥ 64 R	≥ 64 R	≥ 64 R	≥ 64 R	≥ 16 R	2 I	256 R	16 I	≥ 320 R	64 R
KP57	Patient room 02 (Floor)	≥ 32 R	≥ 128 R	≥ 64 R	≤ 4 S	≥ 64 R	≥ 64 R	≥ 16 R	≥ 4 R	128 R	4 S	40 S	≥ 256 R

*Note:* All MIC values are highlighted as numerical values with units µg/mL.

Abbreviations: AMC, Amoxicillin‐clavulanic acid; C, Chloramphenicol; CAZ, Ceftazidime; CIP, Ciprofloxacin; CTX, Cefotaxime; CZ, Cefazolin; F, Nitrofurantoin; FOS, Fosfomycin; FOX, Cefoxitin; GM, Gentamicin; I, Intermediate; R, Resistant; S, Susceptible; SXT, Trimethoprim‐sulfamethoxazole; TZP, Piperacillin‐tazobactam.

### 3.2 Resistome Analysis

WGS analysis revealed the presence of 22 genes conferring resistance to various antibiotic classes (Table 2). The extended‐spectrum β‐lactamase (ESBL) gene *bla*_CTX‐M‐15_, conferring resistance to third‐generation cephalosporins cefotaxime and ceftazidime was observed in all isolates (MIC ≥ 64 µg/mL) along with the broad‐spectrum β‐lactamase gene *bla*_TEM‐1_ (Tables 1 and 2). All isolates carried the *bla*_SHV‐1_, while KP55 possessed *bla*_SHV‐11_. The *bla*_DHA‐1_ conferred phenotypic resistance to cefoxitin in KP55, with a MIC of ≥ 64 µg/mL, unlike the other isolates, which were sensitive (MIC ≤ 4: µg/mL) (Table 1).

**Table 2:** Distribution of antibiotic resistance and virulence genes in K. pneumoniae isolates.

AMR and virulence genes			KP32 (ST13)	KP37 (ST13)	KP57 (ST13)	KP55 (ST8932)
Antibiotic resistance genes	β‐lactam	*bla*_CTX‐M‐15_	+	+	+	+
		*bla*_TEM‐1_	+	+	+	+
		*bla*_SHV‐1_	+	+	+	−
		*bla*_SHV‐11_	−	−	−	+
		*bla*_DHA‐1_	−	−	−	+
		*bla*_OXA‐1_	+	+	+	−
	Quinolone	*qnrS1*	+	+	+	−
		*qnrB4*	−	−	−	+
	Phenicol/Quinolone	*oqxA*	+	+	+	+
		*oqxB*	+	+	+	+
	Aminoglycoside/Quinolone	*aac(6')‐Ib‐cr5*	+	+	+	−
	Aminoglycoside	*aac(3)‐IIe*	+	−	+	−
		*aac(3)‐IId*	+	−	−	+
		*aph(6)‐Id*	+	+	+	+
		*aph(3”)‐Ib*	+	+	+	+
	Sulfonamide	*sul1*	−	−	−	+
		*sul2*	+	+	+	+
	Trimethoprim	*dfrA14*	−	−	−	+
	Fosfomycin	*fosA5*	+	+	+	+
	Phenicol	*catB3*	+	+	+	−
	Efflux pump	*emrD*	+	+	+	+
		*kdeA*	+	+	+	+
Disinfectant resistance gene	Efflux pump	*qacE*	−	−	−	+
Virulence genes	yersiniabactin (Ybt) iron uptake system	*ybt‐irp*	+	+	+	−

Resistance to quinolones was seen due to the presence of the *qnr*, *oqxA, oqxB* and *aac(6')‐Ib‐cr5* genes. The *oqxAB* efflux pump genes responsible for low to intermediate resistance to fluoroquinolones were observed in all isolates. The *aac(6ʹ)‐Ib‐cr5* gene, which confers dual resistance to ciprofloxacin and aminoglycosides was detected in KP32, KP37 and KP57, where it was co‐occurred with *qnrS1*. On the other hand, KP55, which lacked the *aac(6ʹ)‐Ib‐cr5*, but carried the *qnrB4* gene explaining a resistance phenotype to ciprofloxacin in KP32, KP37 and KP57 (MIC ≥ 4 µg/mL) and an intermediate resistance phenotype in KP55 (MIC = 2 µg/mL) (Table 1).

For aminoglycoside resistance genes, we noted the presence of the phosphotransferase genes *aph(6)‐Id* and *aph(3”)‐Ib*, in all isolates, and the aminoglycoside acetyltransferase genes, *aac(3)‐IIe* and *aac(3)‐IId*, were present in two isolates each. As observed by antimicrobial susceptibility results, these genes conferred resistance to gentamicin in all isolates (MIC ≥ 16 µg/mL) (Table 1).

Among the other resistance genes, we identified *sul1* and *sul2* in KP55, which confer resistance to sulfonamides, and*dfrA14*, which confers resistance to diaminopyrimidines. These genes confer high‐level resistance to trimethoprim‐sulfamethoxazole in KP55, with a MIC value of ≥ 320 µg/mL (Table 1). *sul2* was also present in KP32, KP37 and KP57, but *sul1* and *dfrA14* were absent in these isolates. However, a resistance phenotype to trimethoprim‐sulfamethoxazole was observed in KP37(MIC = 80 µg/mL), whereas the other two isolates were sensitive (MIC = 40 µg/mL) (Table 1). Fosfomycin resistance phenotype (MIC = 128–256 µg/mL)was due to the presence of the *fosA5* gene in all isolates (Tables 1 and 2). The chloramphenicol acetyltransferase *catB3* gene, which confers resistance to chloramphenicol, was present only in KP32, KP37, and KP57 isolates (Table 2) but were found to be susceptible, in contrast to the KP55 strain, which showed intermediate resistance but did not possess the gene (Tables 1 and 2). All isolates harbored the multidrug efflux pump genes *kdeA* and *emrD* (Table 2).

The *qacE* efflux pump gene, which provides resistance to quaternary ammonium compounds, was observed only in KP55 (Table 2).

### 3.3 Virulome Analysis

Kleborate analysis revealed the presence of yersiniabactin‐associated virulence genes *ybt‐irp* in KP32, KP37 and KP57 (Table 2). These genes were located on the *K. pneumoniae* integrative conjugative element variant 4 (ICE*Kp*4). The other siderophores (aerobactin and salmochelin), the hypermucoidy locus *rmpADC* and the genotoxin colibactin, which are associated with invasive infections of *K. pneumoniae*, were all absent in our isolates.

### 3.4 Molecular Epidemiology of *K. pneumoniae*

**Figure 1:**
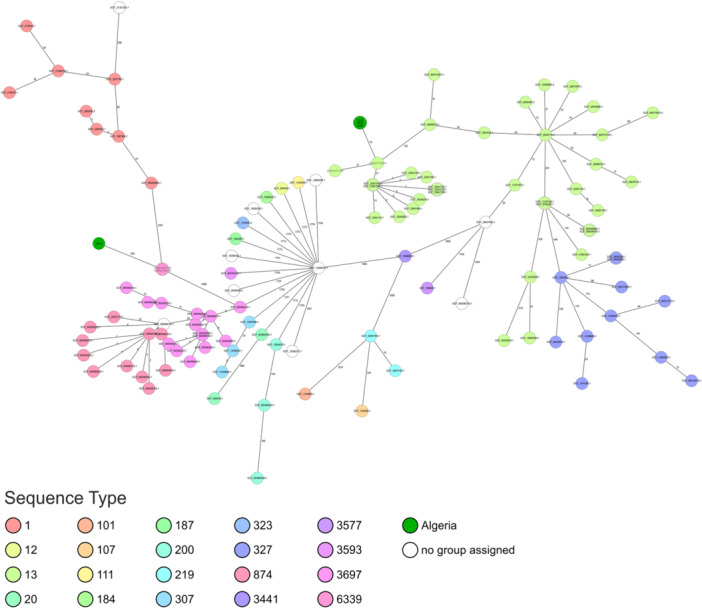
Minimum spanning tree of isolates from bathroom sink (KP55) and floor (KP55) of patient room 2, nurse A's hand (KP32) and a mobile phone of nurse B (KP37) with closely related 112 reference *K. pneumoniae* strains. All Algerian isolates are in dark green circle and each node represents the allelic profile of an isolate (isolate names shown within nodes). The numbers on connecting lines indicate allelic differences between isolates. Node colors correspond to sequence types (see legend).

The WGS results showed a high degree of similarity between KP32, KP37 and KP57 in terms of their resistance genes. The MLST analysis revealed that the three isolates (KP32, KP37 and KP57) are closely related and belong to the same high‐risk ST13 clone (Table 2). The cgMLST showed zero allelic differences between the ST13 isolates KP32, KP37, and KP57. The pairwise comparison of the cgMLST profile between the ST13 isolates and the isolate KP55 showed different allele types at 1991 loci (hereafter referred to as an allelic distance of 1991). KP55 was assigned a new sequence type ST8932, belonging to phylogroup Kp1 and sublineage SL525. The closest sequenced isolate in the constructed collection to the analyzed ST13 isolates was SAMEA7190524, a human blood culture isolate from Germany (Figure 1). This isolate showed an allelic distance of 18 in the cgMLST analysis. The closest isolates in our generated database to KP55 were SAMN18207166 and SAMN27533720, with an allelic distance of 205.

### 3.5 Study Limitations

The study has several limitations that should be acknowledged. These include the relatively small sample size, which may limit the generalizability of the findings; the absence of longitudinal sampling, restricting insights into temporal dynamics; the lack of comparison with clinical isolates, which constrains interpretation of potential transmission pathways; and the use of short‐read sequencing alone, which may limit the resolution of genomic structures such as plasmids and complex resistance regions.

## 4 Discussion

*K. pneumoniae* is an opportunistic pathogen implicated in several neonatal diseases, including respiratory tract infections, urinary tract infections, neonatal sepsis, and meningitis (Harb et al. 2023; Bazaid et al. 2023; Ma et al. 2024; Ali 2024). A growing number of studies have underscored the importance of the hospital environment as a reservoir of pathogenic bacteria carrying antibiotic resistance genes (Kindu et al. 2023; Zahornacký et al. 2022; Nieto‐Rosado et al. 2024; Hanafiah et al. 2024). Our study highlights the importance of studying AMR, virulence genes, and molecular epidemiology of *K. pneumoniae* from the neonatal ward environment in a hospital located in Chlef (Algeria) comparing them with publicly available closely related strains. The WGS data identified the presence of 22 genes encoding resistance to a wide range of antibiotics, including β‐lactams, quinolones, aminoglycosides, fosfomycin, sulfamethoxazole, trimethoprim and chloramphenicol. A multidrug‐resistant phenotype was detected in all isolates. Resistance to β‐lactams, which is considered a serious public health concern, is principally caused by the production of β‐lactamase enzymes. Among the β‐lactamase‐encoding genes, the ESBL gene *bla*_CTX‐M‐15_ was present in all isolates and has been frequently detected in Algeria and worldwide, in the hospital environment and among clinical isolates (Touati et al. 2007; Zenati et al. 2017; Nieto‐Rosado et al. 2024; Randrianirina et al. 2009; Naas et al. 2016; Perez‐Palacios et al. 2023; Rahmani et al. 2023; Nicitra et al. 2025). CTX‐M are extended‐spectrum β‐lactamase enzymes that are becoming increasingly prevalent in both nosocomial and in community settings. The *bla*_CTX‐M‐15_ variant flanked by the insertion element was first identified on plasmids in clinical isolates of *E. coli*, *K. pneumoniae* and *Enterobacter aerogenes* isolated in Batra Hospital and Medical Research Centre in New Delhi around 1999–2000 (Karim et al. 2001). The IS*Ecp1*, believed to provide a strong promoter sequence upstream of the gene was often associated with gene overexpression enhancing the hydrolytic profile of the *bla*_CTX‐M‐15_ β‐lactamase was found in two isolates (KP55 and KP57). The upstream region of *bla*_CTX‐M‐15_ in KP32 and KP37 could not be determined due to poor sequence coverage. It is also important to note that bioinformatic analysis for detecting plasmids in our assemblies predicted *bla*_CTX‐M‐15_ localization on two different types of plasmids (an incL/M plasmid for KP32, KP37, and KP55) and a non‐mobilizable plasmid for KP57, but since the plasmids are not closed, we cannot say for certain whether some of these isolates would aid in the potential transmission and dissemination of plasmids. In our isolates other β‐lactamases encoding genes were detected, including *bla*_TEM‐1_, *bla*_SHV‐1_, *bla*_SHV‐11_ and *bla*_OXA‐1_ which are known to hydrolyze narrow‐ to broad‐spectrum β‐lactams and could potentially act synergistically with ESBLs to enhance resistance. Similar resistance genes have been reported previously in *Enterobacterales* isolated in a Tunisian hospital environment (Dziri et al. 2016). Infections caused by β‐lactam‐resistant *K. pneumoniae* can therefore be challenging to treat, particularly in neonates, and can have a poor outcome (Geleta et al. 2024).

Of the β‐lactam antibiotics tested, cefoxitin sensitivity was observed in most of our isolates, except in KP55, (MIC ≥ 64 µg/mL) which harbored the *bla*_DHA‐1_ gene, which is the main inducible plasmid‐mediated AmpC cephalosporinase (pAmpC) capable of hydrolyzing a wide range of β‐lactams, including cephamycins (Jacoby 2009). In addition to *bla*_DHA‐1_, KP55 also carried the *qnrB4* gene, which can reduce the binding affinity of fluoroquinolones such as ciprofloxacin and levofloxacin to their target enzymes and can spread via plasmids. The other ESBL isolates (KP32, KP37 and KP57) co‐produced*bla*_CTX‐M‐15_, *qnrS1* and *aac(6ʹ)‐Ib‐cr5* genes. Previous studies from Algeria have reported the presence of *qnrS1* in ESBL‐producing *K. pneumoniae* in clinical isolates. (Zemmour et al. 2021) In agreement with our findings, the co‐occurrence of ESBL and quinolone resistance genes in *Enterobacterales* has been reported in several published studies from Algeria (Zenati et al. 2017; Zemmour et al. 2021; Anssour et al. 2014, 2016; Medboua‐Benbalagh et al. 2017) and worldwide (Xiong et al. 2021; Kibwana et al. 2023; Piekarska et al. 2019). In the current study, no mutations were detected in the quinolone resistance‐determining regions of the *gyrA* and *parC* genes.

We identified the presence of aminoglycoside phosphotransferase (*aph*) and aminoglycoside acetyltransferase (*aac*) genes, which confer resistance to aminoglycoside antibiotics (Ramirez and Tolmasky 2017). In addition, all isolates exhibited high resistance to nitrofurantoin and fosfomycin, and KP55 showed very high resistance to trimethoprim‐sulfamethoxazole, with MICs of ≥ 320 µg/mL. While resistance to fosfomycin is due to the presence of fosfomycin‐modifying genes *fosA5* in all isolates, (Mattioni Marchetti et al. 2023) and resistance to trimethoprim‐sulfamethoxazole is conferred by sulfonamide resistance genes *sul1*, *sul2* and trimethoprim resistance gene *dfrA14*, (Sköld 2001) no genes encoding nitrofurantoin resistance were observed in this study. Resistance to this antibiotic could be due to the overexpression of the *oqxA* and/or *oqxB* genes, which were present in all isolates. In a recent study, high resistance to nitrofurantoin was attributed to overexpression of *oqxB* in *K. pneumoniae* mutants with altered *oqxR* (transcriptional repressor of the *oqxAB* efflux pump) (Hussein et al. 2024). In another study, involvement of the OqxAB efflux pump in nitrofurantoin resistance in uropathogenic *K. Pneumoniae* has also been reported (Xu et al. 2019).

MLST analysis of KP55 identified it as a novel strain assigned to ST8932 and as a single‐locus variant (*tonB*) closely linked to ST525 which has been earlier detected in Norway that harbored *bla*_NDM‐1_ + *bla*_OXA‐181_ (Samuelsen et al. 2017). Interestingly, there were no ST525 in the collection of closely related isolates to KP55 and the closest matching isolates to KP55, with an allelic distance of 205, were SAMN18207166 and SAMN27533720, both ST6339 (Figure 1). The isolate SAMN18207166 originated from a patient's blood sample in 2018 in Maryland, Orlando, whereas SAMN27533720 originated from a hospital in the USA (Supporting file 1). It should be noted that KP55 was isolated from a patient's bathroom sink, highlighting the importance of sinks and their drains as reservoirs for antibiotic‐ and disinfectant‐resistant bacteria (Valentin et al. 2021; De Geyter et al. 2017; Fucini et al. 2023; Diorio‐Toth et al. 2023; Anantharajah et al. 2024; Li et al. 2025; Chapuis et al. 2016; Rath et al. 2024). This is in line with previous studies that have found a clonal link between environmental bacteria collected from sinks and sink drains and the clinical environment (Manageiro et al. 2024; De Geyter et al. 2017; Anantharajah et al. 2024; Simões 2022).

Phenotypic and genotypic analyses revealed that KP32, KP37, and KP57 were identical and highly clonal in nature (allelic distance of 0) assigned to the high‐risk ST13 clone. The cgMLST analysis identified SAMEA7190524 as the closest matching isolate to KP32, KP37, and KP57. SAMEA7190524 was initially isolated in 2017 from a blood culture sample in a hospital in Cologne, Germany. An allelic distance of 18 was observed between our ST13 isolates and SAMEA7190524, showing a close association and high similarity between our strains and this hospital‐associated isolate (Figure 1).

It is important to note that the three ST13 isolates originated from distinct sources: KP32 from a nurse's hands, KP37 from another nurse's phone, and KP57 from the floor of patient room 02 the same room where KP55 was recovered from the bathroom sink. This highlights the potential dissemination of the multidrug‐resistant clone ST13 in the neonatal ward. It has previously been reported that bacteria isolated from staff hands were significantly more similar to the microbial communities on environmental surfaces within the healthcare setting than to those on patient hands, likely due to staff movement throughout the facility (Lax et al. 2017). Transmission of ST13 clone to hospitalized neonates is also possible but would require other environmental and clinical isolates and molecular epidemiology analysis, which is difficult in a resource‐limited setting but can indeed provide further information on the spread of clones in a hospital setting. The presence of ST13 has been reported in hospitals across three separate regions of Algeria, on inanimate surfaces, (Zenati et al. 2017) in clinical neonatal isolates and isolates from the pediatric ward (Labid et al. 2023; Belbel et al. 2014). Another study, conducted on isolates from various origins and regions in Algeria, described the ST13 clone as endemic in the province of Béjaïa, Algeria (Mairi et al. 2019). ST13 isolates have also been reported in several other countries, such as Morocco, (Perez‐Palacios et al. 2023) Portugal, (Simões 2022; Mendes et al. 2022; Spadar et al. 2022) South Africa, (Marais et al. 2024) Russia (Fursova et al. 2020) and France (Marcade et al. 2013) highlighting the hospital environment as a reservoir of highly pathogenic lineages.

## 5 Conclusion

This study emphasizes the critical role of the hospital environment as a reservoir for AMR pathogens. Although the *K. pneumoniae* isolates examined in this study had a low virulence score, they exhibited a multi‐drug‐resistant profile, conferring resistance to major antibiotic classes including β‐lactams, quinolones, and aminoglycosides, which are widely used to treat *K. pneumoniae* infections. Hence, other alternative therapeutic options, and urgent need for antimicrobial stewardship is needed for infection prevention and control which can slow down antimicrobial resistance. This study also reports ST8932 for the first time, along with three clonal isolates of the high‐risk ST13 clone from different locations within the same hospital, suggesting their potential to spread and cause life‐threatening infections in neonates. The rise of multidrug‐resistant infections is not only due to the selective pressure exerted by antibiotic use in medical treatment, but also to the interplay of factors that influence molecular evolution. These may include both horizontal and vertical transmission, as well as interactions between human and hospital environment reservoirs within the healthcare setting, which are critical for understanding emerging transmission pathways. More surveillance strategies and WGS approaches can provide us with a better picture and help us in source tracking by estimating full potential of resistant bacteria in different environments. Effective antimicrobial stewardship and proper hand hygiene are crucial for reducing bacterial contamination in hospital settings and for preventing healthcare‐associated infections among newborns by maintaining antibiotic effectiveness, which is rightly considered one of the most groundbreaking medical innovations of our era.

## Author Contributions

**Meriem Derkaoui:** conceptualization, investigation, funding acquisition, visualization, validation, methodology, resources, writing – original draft. **Martin Alexander Fischer:** investigation, methodology, software, resources, data curation, formal analysis. **Mohammed Sebaihia:** conceptualization, investigation, funding acquisition, visualization, project administration, resources. **Rachida Namoune:** investigation, visualization, project administration, resources. **Fatiha Bouheudjeur:** investigation, validation, formal analysis. **Meryem Beloukarif:** investigation, validation, formal analysis. **Bakhta Madaoui:** funding acquisition, investigation, validation, formal analysis. **Bruno Silvester Lopes:** conceptualization, investigation, writing – review and editing, resources, supervision, visualization, formal analysis, methodology.

## Funding

The authors have nothing to report.

## Ethics Statement

The authors have nothing to report.

## Conflicts of Interest

The authors declare no conflicts of interest.

## Supporting information


**Supporting File 1**: Accession numbers for 112 unique isolates including 4 Algerian isolates that were used to construct the cgMLST‐based minimal spanning tree using Ridom SeqSphere+.

**Supporting File 2**: Reporting of genome assembly quality metrics for whole‐genome sequencing data of *K. pneumoniae* isolates.

## Data Availability

All data analyzed are freely available, and the accession numbers can be obtained through the NCBI website https://www.ncbi.nlm.nih.gov/ with information from the Supporting Files.
